# Laryngeal papillomatosis: morphological study by light and electron microscopy of the

**DOI:** 10.1016/S1808-8694(15)30600-5

**Published:** 2015-10-18

**Authors:** Regina Helena Garcia Martins, Norimar H. Dias, Elisa Aparecida Gregório, Mariângela Alencar Marques, Márcia Guimarães da Silva, João Manuel Grisi Candeias

**Affiliations:** 1 Livre-Docente Professor, Otorhinolaryngology Discipline, Botucatu Medical School - UNESP. Adjunct Professor, Otorhinolaryngology Discipline, Botucatu Medical School - UNESP.; 2 Physician and Graduate Doctoral Student, Otorhinolaryngology Discipline, Botucatu Medical School - Unesp.; 3 Full Professor, Morphology Department, Institute of Biosciences - UNESP - Botucatu.; 4 Adjunct Professor, Pathology Department, Botucatu Medical School - UNESP.; 5 Adjunct Professor, Pathology Department, Botucatu Medical School - UNESP.; 6 Adjunct Professor, Microbiology and Immunology Department, Institute of Biosciences - UNESP, Botucatu. Universidade Estadual Paulista Julio de Mesquita Filho - Faculdade de Medicina de Botucatu (Botucatu Medical School).

**Keywords:** larynx, microscopy, papillomatosis, ultrastructure

## Abstract

Laryngeal papillomatosis is the most frequent benign neoplasia in children. It is caused by HPV 6 and 11. The lesions are exophytic and highly recurrent, compromising the airway mucosa, mainly the larynx. Study design - clinical prospective.

**Aims:**

to show morphologic alterations of the epithelium (light and electron microscopy) in the HPV-6 lesions.

**Methods:**

specimens of laryngeal lesions obtained during surgery of four children (1 male, 3 female) were submitted to HPV typing (PCR), light microscopy and electron microscopy.

**Results:**

in all specimens, HPV type 6 was found. Epithelial projections were found by electron microscopy with superficial cells in desquamation. Light microscopy showed exophytic projections of the keratinized stratified squamous epithelium overlying a fibrovascular core. Koilocytes (vacuolated cells), suggesting the viral infection by HPV, were identified. No alterations were seen in the basement membrane and corion. Ultraestrutural analysis showed vacuolated cells with clear cytoplasmic inclusions, intercellular injuries and widening intercellular spaces.

**Conclusions:**

morphologic alterations of the epithelium in the HPV-6 lesions are superficial, and additional studies including the others HPV types are needed to show the more aggressive and extensive aspect of the disease.

## INTRODUCTION

Laryngeal papillomatosis is a benign neoplasm characterized by exophytic proliferative lesions of connective tissue covered by epithelium. It usually starts in the commissure and anterior third of the vocal folds, and may eventually affect all of the larynx, and even the trachea, bronchii and lung parenchyma in some cases. Lesions are generally multiple, rarely solitary.^1^, ^2^, ^3^

The juvenile form affects children in their first years of life; symptoms include fixed hoarseness that becomes progressive dyspnea an eventually dramatic clinical pictures of respiratory discomfort and stridor. In these cases, there are multiple vegetating confluent lesions occluding the glottic lumen, often extending to supra- and subglottic structures. The adult form affects adolescents and adults; in these cases there are fewer lesions that are focal and less recurring, but the malignancy potential is increased.^3^, ^4^

Ullman was the first to describe the viral etiology of laryngeal papillomatosis, in 1923.^5^ The virus itself, the HPV (human papillomatosis virus), was demonstrated by electron microscopy, in situ hybridization techniques and the polymerase chain reaction (PCR).^6^, ^7^, ^8^ As these techniques developed, different subtypes of papillomavirus were identified; there are currently over 60 of these subtypes. Laryngeal papillomatosis is the most important manifestation of HPV laryngeal infection, mostly involving the subtypes 6 and 11, which are also involved in genital condylomas.^9^ On the other hand, subtypes 16 and 18 are involved in laryngeal neoplasms and in uterine cervical carcinomas.^10^, ^11^

Notwithstanding these advances in HPV DNA studies, its transmission mode remains unclear. HPV is positive in the airways of neonates and in maternal genital condylomas, which suggests that it is transmitted in the birth canal, from mothers with genital condylomas.^12^ Occasionally, however, HPV is positive in newborns of mother with no history of genital condylomas, suggesting the possibility of latent or subclinical products, which may explain recurrences after a long time period of disease remission.^13^

Some studies have found HPV in benign laryngeal lesions such as polyps, Reinke's edema, leukoplasia, lichen planus, and even in the normal mucosa of the mouth.^14^, ^15^ Thus, the presence of HPV in the upper airway mucosa may not to be the determining factor for disease. Other simultaneous factors have been looked at, such as immunodeficiency and associated infection, particularly the herpes virus, the Epstein Barr virus (EBV) and the cytomegalovirus (CMV).16,17 Some authors have tried to demonstrate a possible genetic susceptibility that would lead or not to the development of laryngeal papillomatosis as a result of epithelial HPV infection.^18^, ^19^

Laryngeal papillomatosis is still a major challenge to pediatricians, infectologists, immunologists and otorhinolaryngologists, even with recent advances in diagnosis. New research on HPV has added precious information to help elucidate the mysteries around this disease. However, there have been few morphological studies in this area.

The purpose of this study was to analyze morphological alterations seen in laryngeal papillomatosis caused by HPV subtype ^6^.

## MATERIAL AND METHOD

The Research Ethics Committee of the institution in which the investigation was done approved the study (protocol number 411/2006). Specimens collected during surgery of four children with a diagnosis of laryngeal papillomatosis were analyzed, and these children were monitored in the Speech Therapy and Voice Disorder Unit where the study was done. The children included in this study consisted of one boy and three girls; three of them were aged between 3 and 6 years, and one was aged 15 years. All children presented with hoarseness and respiratory discomfort, mostly upon significant effort; all had already undergone various surgical procedures in the past. None of their mothers reported the presence of papillary lesion or prior treatment of condyloma.

At the time of surgery, fragments of laryngeal lesions were reserved for light and electron (transmission and scanning) microscopy and for defining the HPV subtype using molecular techniques.

Light microscopy - fragments were first fixated in 10% formaldehyde for at least 48 hours, then included in paraffin, sectioned, and hematoxyllin-eosin (HE) stained. The specimens were also PAS (periodic acid-Schiff) stained for clearer imaging of the basement membrane. Next, the histological slides were examined under light microscopy at different magnification, and the images were taken using a digital camera (Leica Q-Win software version 3.1).

Electron microscopy - scanning and transmission electron microscopy were done on new fragments fixated in 2.5% glutaraldehyde, according to the procedures described by the Electron Microscopy Center of the Bioscience Institute (Centro de Microscopia Eletrônica do Instituto de Biociencias). Following preparation, specimens were examined and photographed under scanning electron microscopy (SEM 515, Philips - Holland), at 15KV. The exposed surface was tracked quadrant by quadrant, and then photographed at increasing magnification. Sections were also examined and photographed under transmission electron microscopy (model EM 301, Philips AG - Holland).

The results of morphological analysis were presented descriptively.

HPV typing - viral DNA was extracted using a CTAB (cetyltrimethylammonium bromide) solution and tested with the polymerase chain reaction (PCR). Viral DNA was transferred to the membrane using the Dot Blot technique, for typing. Marked probes from the kit AlkPhos Direct (Amersham) were used to detect the types of HPV. The amplification efficiency was monitored by 1.5% agarose gel reaction electrophoresis (Gibco BRL). The size of amplified products were compared with the 123 pb standard (Gibco BRL) and photographed under ultraviolet transillumination.

## RESULTS

**Figure 1:**
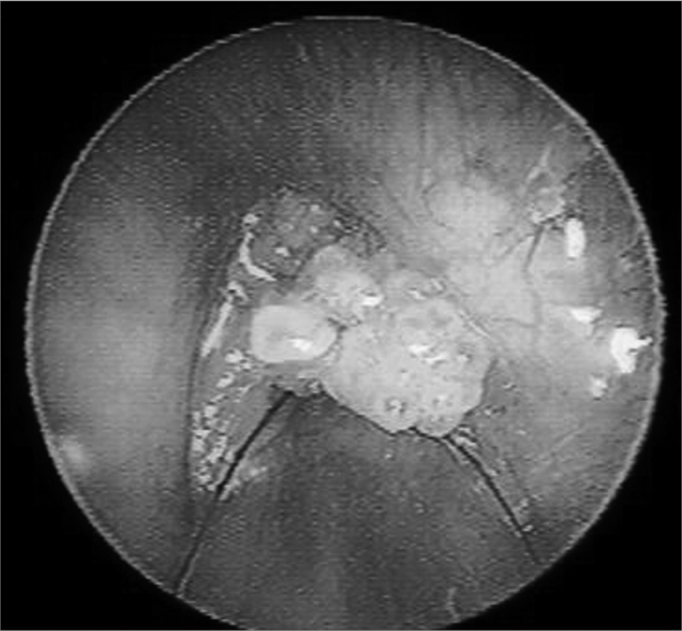
Endoscopy of the larynx. Note the pale exophytic growths on both vocal folds.

**Figure 2:**
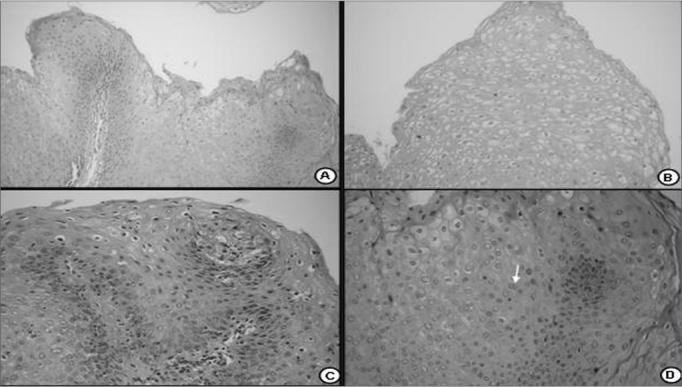
Histological section of a squamous papilloma (HE 40X); b - numerous koilocytes (HE 200X); c - detail of cytopathic effects of the virus; many cells with perinuclear vacuolization and parakeratosis (HE 400X); d - binucleated koilocytes (seta- HE. 400X).

In this study all of the specimens that were examined belonged to the HPV subtype 6. The vocal fold mucosa that was affected by HPV was covered by pale exophytic papilliferous growths located mostly in the anterior glottic region (Figure 1). Light microscopy of HE-stained slides showed exophytic lesions covered by hyperplasic epithelium with papillomatosis, acanthosis and hyperparakeratosis. Keratinocytes had irregular hyperstained nuclei, with a velvety chromatin and a clear and well-defined perinuclear halus, which characterized koilocytes. Mitosis was seen rarely, but binucleation was frequent (Figure 2).

**Figure 3:**
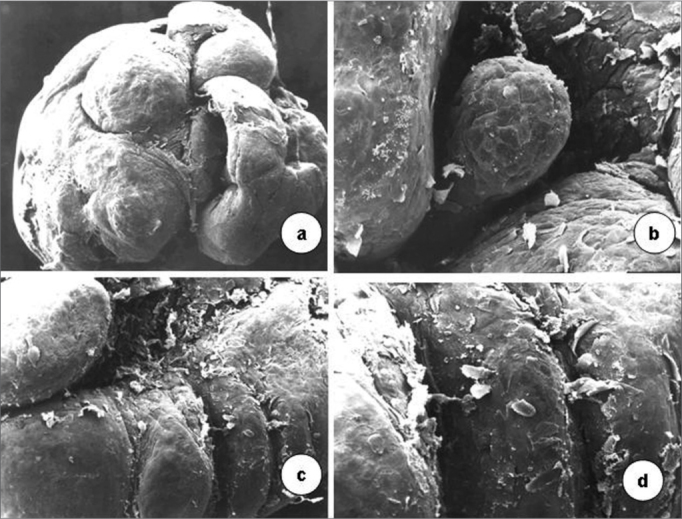
Surface of the laryngeal mucosa involved by the type 6 papilloma virus. Pale mucosa with differently sized growths projecting from the surface; many desquamating cells (scanning electron microscopy; a-50X; b-1.000X; c-2.500X; d-200X).

Scanning electron microscopy revealed that vocal folds were covered by epithelial projections with many desquamating superficial cells. The growths varied in size; similar smaller growths appear among larger ones. Cell desquamation appeared more intense in certain regions (Figure 3).

**Figure 4:**
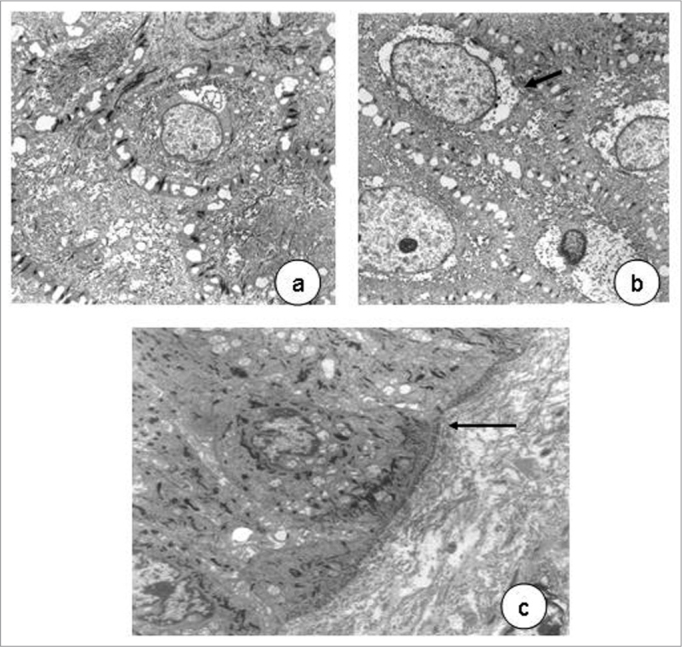
In a, epithelial cells with decondensed chromatin, evident nucleoli (3,250X); widened cell junctions. In b, perinuclear vacuolization (arrow, 11,000X)). In c, detail of a normal basement membrane (arrow, 6,300X) - transmission electron microscopy.

Transmission electron microscopy showed that cell nuclei contained decondensed chromatin and evident nucleoli. Various organelles were found in the cytoplasm, especially mitochondria, suggesting intense cell metabolism. Usually interdigitated intercellular spaces were widened, distorting the desmossomal structure. Perinuclear vacuolization was evident in light and transmission electron microscopy (Figure 4).

## DISCUSSION

Papillomatosis may present with severe involvement of the laryngeal mucosa; there may be recurring lesions in the vocal folds, the pharynx and the trachea. In extreme cases this may cause acute respiratory failure. This condition is the most common laryngeal benign neoplasm in children. Although it was described decades ago, its pathophysiology and treatment have been the target of much research.^20^, ^21^

HPV is a DNA virus that parasitizes epithelial cell nuclei. It is difficult to identify the virus with light and electron microscopy, as our study showed. Molecular biology is required to confirm the parasite and establish its subtype. In our study, a marked epithelial change that was seen both under light and transmission electron microscopy, was perinuclear vacuolization, which is typical of HPV infection.^22^ The Brazilian Consensus on HPV^23^ includes this finding as a major criterion among the cytological findings of HPV infection. Light and electron microscopy revealed that the basement membrane was intact, and that the chorion and the laryngeal muscles were not involved. These findings underline the superficial and non-infiltrating nature of lesions caused by the HPV-6.

All HPV types taken from laryngeal lesion in our sample belonged to the subtype 6. It is known that HPV-6 and 11 are frequent in children and teenagers, and that they do not malignize despite frequent recurrences. On the other hand subtypes 16 and 18, which may occasionally be found in laryngeal papillomatosis, do have a malignant potential. Padayachee and Prescott^24^ have suggested that the HPV-6 tends to be more aggressive than the HPV-11; most studies, however, have shown that disease by subtype 11 is more aggressive, with earlier and more frequent recurrences.^25^, ^26^, ^27^ Wiatrak et al.^28^ studied 73 patients with laryngeal papillomatosis and found the HPV-6 in 53.5% of cases, the HPV-11 in 39.7% of cases, and both subtypes in 6.9% of cases.

There have been few morphological studies showing the ultrastructural changes caused by each HPV subtype. Most studies do not even inform the HPV type. This study of the HPV-6 morphology enabled us to conclude that epithelial changes are superficial, and that deeper structures such as the basement membrane and the adjacent chorion are preserved. However, the more aggressive behavior of subtypes 11, 16 and 18 may open the possibility for identifying deeper epithelial changes, as well as atypias, since subtypes 16 and 18 have been related to airway malignancies.

## CONCLUSION

Morphological changes caused by the HPV-6 demonstrate its non-invasive nature. Additional morphological studies are needed to investigate the ultrastructural alterations caused by other HPV subtypes that are considered as more aggressive.
